# Fructose Rich Diet-Induced High Plasminogen Activator Inhibitor-1 (PAI-1) Production in the Adult Female Rat: Protective Effect of Progesterone

**DOI:** 10.3390/nu4081137

**Published:** 2012-08-22

**Authors:** Daniel Castrogiovanni, Ana Alzamendi, Luisina Ongaro, Andrés Giovambattista, Rolf C. Gaillard, Eduardo Spinedi

**Affiliations:** 1 Neuroendocrine Unit, IMBICE (CONICET-CICPBA), 1900 La Plata, Argentina; Email: dcastrogiovanni@imbice.org.ar (D.C.); anitaalzamendi@yahoo.com.ar (A.A.); ongaroluisina@imbice.org.ar (L.O.); agiovamba@imbice.org.ar (A.G.); 2 Division of Endocrinology, University Hospital (CHUV), CH 1011 Lausanne, Switzerland; Email: rolf.gaillard@chuv.ch

**Keywords:** high carbohydrate diet, glucose tolerance, insulin, adipokines, allostasis

## Abstract

The effect of progesterone (P4) on fructose rich diet (FRD) intake-induced metabolic, endocrine and parametrial adipose tissue (PMAT) dysfunctions was studied in the adult female rat. Sixty day-old rats were i.m. treated with oil alone (control, CT) or containing P4 (12 mg/kg). Rats ate Purina chow-diet *ad libitum* throughout the entire experiment and, between 100 and 120 days of age drank *ad libitum* tap water alone (normal diet; CT-ND and P4-ND) or containing fructose (10% w/v; CT-FRD and P4-FRD). At age 120 days, animals were subjected to a glucose tolerance test or decapitated. Plasma concentrations of various biomarkers and PMAT gene abundance were monitored. P4-ND (*vs.* CT-ND) rats showed elevated circulating levels of lipids. CT-FRD rats displayed high (*vs.* CT-ND) plasma concentrations of lipids, leptin, adiponectin and plasminogen activator inhibitor-1 (PAI-1). Lipidemia and adiponectinemia were high (*vs.* P4-ND) in P4-FRD rats. Although P4 failed to prevent FRD-induced hyperleptinemia, it was fully protective on FRD-enhanced plasma PAI-1 levels. PMAT leptin and adiponectin mRNAs were high in CT-FRD and P4-FRD rats. While FRD enhanced PMAT PAI-1 mRNA abundance in CT rats, this effect was absent in P4 rats. Our study supports that a preceding P4-enriched milieu prevented the enhanced prothrombotic risk induced by FRD-elicited high PAI-1 production.

## 1. Introduction

Fructose corn syrup has become a popular sweetener used in soft drinks and many other products of modern, massive daily consumption. In countries like the USA, estimated annual *per capita* fructose consumption rose from 64 g/day during the 1970s to 81 g/day at the end of the 1990s [1], with an additional increase in fructose intake (2.5 g/day) resulting from increased fruit and vegetable consumption. It has been postulated that such increased consumption could contribute to the current epidemics of human obesity, Type 2 diabetes mellitus and metabolic syndrome (MS) [2]. Several studies demonstrated that administration of a fructose-rich diet (FRD) to normal rats of either sex induces several features of the human phenotype of MS [3,4,5]. Regarding the mechanisms whereby FRD-intake induced MS, it has been suggested that the high intake of fructose increases the production of reactive oxygen species (ROS), thus enhancing general and adipose tissue (AT) oxidative stress (OS) [6]. As a consequence, the enlarged abdominal adipocytes of FRD fed rats overproduce leptin [7,8]. 

It has been reported that sex steroids modulate OS and particularly progesterone (P4) has been postulated as a positive instigator of OS [9], probably by an indirect mechanism secondary to the activation of glucocorticoid receptors [10]. It is openly accepted that AT-derived adipokines regulate gonadal function, and that sex steroids such as testosterone [5,11] modulate white adipocyte endocrine function; however, little is known about potential P4 activity on AT function. Although lipogenic P4 activity has been reported in isolated adipocytes [12,13], and *in vivo* in the normal female rat [14], P4 has also been identified as a stimulator of insulin degradation at the pancreatic level [15]. Contrary to the effect of androgens, P4 did not prevent pancreatic cell death [16]. P4 could also act as an inhibitor of glucose-stimulated insulin secretion from pancreatic islets [17]. P4 treatment, either alone or in combination with estradiol (E2), modulates insulin sensitivity [18,19] without affecting glucose transporter (GLUT)-4 activity [18]. Moreover, other studies indicate that P4 impairs insulin sensitivity by antagonizing insulin action [20] and that this steroid is able to counteract the hyperglycemic and lipolytic effects of glucagon in the post-prandial state [21].

The aim of the present study was to explore whether a previous and transient P4-rich endogenous environment in the early post-pubertal female rat could modulate subsequent development of several metabolic, endocrine and adipose tissue dysfunctions induced by the intake of a FRD. 

## 2. Experimental Section

### 2.1. Animals and Experimental Design

The estrous cycle of individually housed, adult Sprague-Dawley rats was daily checked for a week prior experimentation, thus rats do not displaying a complete estrous cycle were excluded from experimentation. Animals were kept in a temperature (21 °C)—and light (lights on 07:00 to 19:00 h)—controlled room with free access to Purina Rat Chow (normal diet; ND) and water. The sex steroid-priming protocol used in this study was previously developed in our laboratory for testing the effect of early intervention with a sex steroid (testosterone) on later metabolic-endocrine functions in the early post-pubertal female rat [11]. Briefly, randomly cycling rats were injected intramuscularly (i.m.) with 100 μL of sterile corn oil alone (control, CT; *n* = 20 animals) or containing Progesterone (12 mg/kg, P4; *n* = 24 rats) on day 60 of age. Rats were left undisturbed for 40 days (between age days 60 and 100). Thereafter, all rats were switched to drinking a fructose 10% (w/v) solution in water (FRD) while eating *ad libitum* Purina rat chow for 21 days (between age days 100 and 120) [5]. Fresh fructose solution was provided daily and, energy intake and body weight were recorded daily throughout the study. Animals were either decapitated between 8:00 and 10:00 h on day 120 of age in non-fasting conditions or after overnight fasting subjected to an i.v. glucose-tolerance test. Parametrial adipose tissue (PMAT) pads were dissected from rats sacrificed in non-fasting condition, weighed and kept frozen (at −80 °C) until processed. Additionally, for the purpose of avoiding any confusing effect of P4 treatment by itself, oil (CT)- and P4-treated rats were provided drinking water and normal chow diet (ND) *ad libitum* throughout both experimental periods (between age 60 and 120 days; these groups were abbreviated as CT-ND and P4-ND; *n* = 12–14 rats per group) (see Table 1). Experiments complied with international regulations concerning the ethical use of animals, and were also approved by our Institutional Animal Care Committee. 

**Table 1:** Summary of the experimental design.

Treatment (i.m. on age 60 days)	Diet (100–120 days of age)	Group (abbreviation)
Vehicle	Normal	CT-ND
P4	Normal	P4-ND
Vehicle	Fructose Rich	CT-FRD
P4	Fructose Rich	P4-FRD
	Experimentation (on day 120 of age)	

### 2.2. Peripheral Metabolite Measurements

Plasma glucose (Wiener Argentina Lab.), triacylglycerol (TG; Wiener), total cholesterol (Wiener) and non-esterified fatty acid (NEFA; Randox Laboratories Ltd., UK) levels were measured using commercial kits. Plasma leptin [22], insulin [23], corticosterone [24], E2 [24] and testosterone (T) [24] concentrations were determined by specific radioimmunoassays (RIAs) developed in our laboratories, while those of P4 were measured using a commercial RIA kit (Immunotech Laboratories, Inc. Glendale, CA, USA). Coefficients of variation (CVs) intra- and inter-assay for all RIAs, ranged between 4% and 6% and 8% and 12%, respectively. Plasma levels of other adipokines were assayed by following the recommendations of the respective retailers of different ELISA kits (Linco Research, Cat. #EZRADP-62K for adiponectin, ADIPOQ; and American Diagnostica Inc., CT, USA, IMUCLONE Cat. #601 for plasminogen activator inhibitor factor-1, PAI-1). 

### 2.3. Intravenous Glucose Tolerance Test (i.v.-GTT)

Metabolic responses to a high i.v. glucose load (2 g/kg BW) were measured in rats under light ketamine anesthesia. Rats were bled before (sample time 0) and several times (5, 15, 30, 60 and 90 min) post-glucose administration [25]. Plasma samples were kept frozen (−20 °C) until determination of glucose and insulin concentrations as described above.

### 2.4. Parametrial Adipose Tissue RNA Isolation and Real-Time Quantitative PCR

Total RNA was isolated from PMAT pads of rats from different groups by the single-step, acid guanidinium isothiocyanate-phenol-chloroform extraction method (Trizol; Invitrogen, Life Tech., USA; cat. #15596-026) [26]. One µg of total RNA was reverse-transcripted using random primers (250 ng) and Superscript III Rnase H-Reverse Transcriptase (200 U/μL Invitrogen, Life Tech, USA; cat #18989-093). Primers applied: *β-actin* (ACTB) (R): 5′-ACCCTCATAGATGGGCACAG-3′, (F): 5′-AGCCATGTACGTAGCCATCC-3′ (115 pb) (GenBank Accession Number (GBAN): NM_031144); *LEP* (R): 5′-CTCAGCATTCAGGGCTAAGG-3′, (F): 5′-GAGACCTCCTCCATCTGCTG-3′ (192 pb) (GBAN: NM_013076); *ADIPOQ* (R): 5′-TCTCCAGGAGTGCCATCTCT-3′, (F): 5′-AATCCTGCCCAGTCATGAAG-3′ (159 pb) (GBAN: NM_144744); *PAI-1* (R): 5′-TCTCCAGGGGCCCTCTGAGGT-3′, (F): 5′-TGCCCCTCTCCGCCATCACC-3′ (141 pb) (GBAN: NW_047370); *IRS-1* (R): 5′-ACGGTTTCAGAGCAGAGGAA-3′, (F): 5′-TGTGCCAAGCAACAAGAAAG-3′ (176 pb) (GBAN: NM_012969); and *GLUT-4* (R): 5′-TGGACGCTCTCTTTCCAACT-3′, (F): 5′-GCTTCTGT-TGCCCTTCTGTC-3′ (166 pb) (GBAN: NM_012751). Two microliters of the reverse transcription mix were amplified with QuantiTect Syber Green PCR kit (Qiagen, cat. #204143) containing 0.5 μM of each specific primer using a LightCycler Detection System (MJ Mini Opticon, Biorad). PCR efficiency was ~1. Cycle thresholds (Ct) were measured in separate tubes by duplicate. Identity and purity of the amplified product were checked by electrophoresis on agarose mini-gels and the melting curve was analyzed at the end of amplification. Values of the differences between the cycle threshold (Ct) were calculated in every sample for each gene of interest as followed: Ct (gene of interest) − Ct (reporter gene). *β-actin*, whose mRNA levels did not differ comparing control to test groups, was the reporter gene. Relative changes in expression level of one specific gene (ΔΔCt) were calculated as ΔCt of the test group minus ΔCt of the control group and then presented as 2^−ΔΔCt^.

### 2.5. Statistical Analysis

Data (means ± SEM) were analyzed by ANOVA (one-factor (treatment) or two-factor (treatment × diet) when appropriate) followed by Fisher’s test. The nonparametric Mann–Whitney test was used to analyze data from PMAT mRNA expression [27]. *p* values lower than 0.05 were considered statistically significant. 

## 3. Results

### 3.1. Effect of Diet on Body Weight, Parametrial Adipose Tissue Mass and Energy Intake in CT and P4-Primed Rats

**Figure 1:**
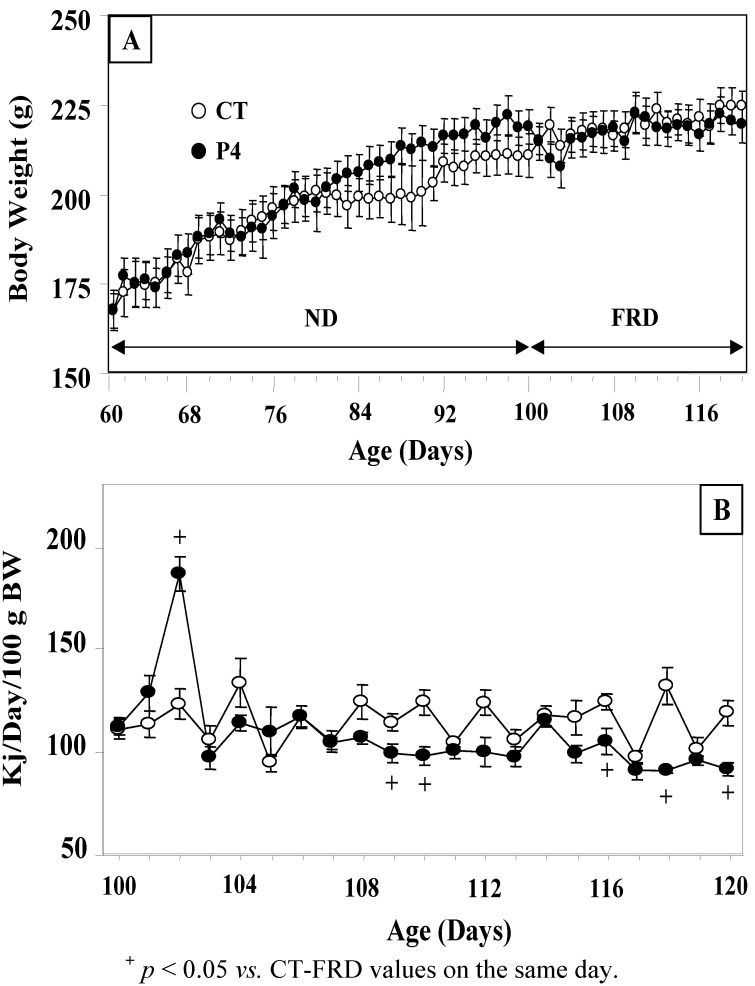
Daily body weight (**A**) values in control (CT) and P4-treated rats fed a normal diet (ND) (between age days 60 and 100) or a fructose rich diet (FRD) (between days 100 and 120). The daily energy intake over a 3 week-period (between days 100 and 120) of feeding rats FRD is also shown (**B**). Values are means ± SEM, *n* = 6–7 animals per group.

Figure 1A shows that P4 treatment did not modify individual body weight (BW) in rats normally nourished (ND) (between age days 60 and 100). Moreover, P4 treatment did not modify rat BW when fed a FRD (between age days 100 and 120). On the experimental day, BW (224.61 ± 4.19 and 219.33 ± 5.18 g) and PMAT mass (1.64 ± 0.09 and 1.79 ± 0.11 g/100 g BW) were similar (*p* > 0.05) in CT-FRD and P4-FRD rats (*n* = 6/7 rats per group).

The average rat daily energy intake was not influenced by P4 treatment (between age days 60 and 100; in kJ/day/100 g BW; 105.95 ± 5.17 in CT and 104.47 ± 6.05 in P4 rats). Thereafter, the 21-day average daily energy intake during the FRD administration period (between days 100 and 120 of age) to rats was also similar in the two groups (in kJ/day/100 g BW; 114.68 ± 3.44 in CT-FRD and 107.71 ± 4.59 in P4-FRD rats); being energy intake statistically similar (*p* > 0.05) in both periods regardless of treatment and diet; as well as after compared the 60 day average-period (in kJ/day/100 g BW, 108.11 ± 1.73 in CT and 106.53 ± 1.88 in P4 rats; *p* > 0.05). Interestingly, P4-primed rats displayed some hedonic behavior for the FRD that only lasted for few days (Figure 1B). Thereafter, P4-FRD animals recovered a normal daily energy intake, and even showed a lower daily energy intake (*vs.* CT-FRD rats) on a few days of the FRD intake period, thus compensating for a normal 21-day average energy intake (Figure 1B). Nevertheless the 21-day average daily fluid intake was similar in both groups (in mL/day/rat; 36.8 ± 1.2 in CT-FRD and 35.1 ± 2.1 in P4-FRD rats; *p* > 0.05).

### 3.2. Effect of FRD Intake on Peripheral Biomarkers in Normal and P4-Primed Rats

We found that non-fasting peripheral levels of glucose, insulin, progesterone, total cholesterol, corticosterone, estradiol and testosterone were similar in CT and P4 rats regardless of the diet administered (Table 2). Conversely, P4 treatment (P4-ND rats) already induced a significant (*p* < 0.05 *vs.* CT-ND values) increase in plasma concentrations of TG and NEFA (Table 2). Although plasma total cholesterol concentrations were not influenced by FRD intake in CT rats, it was significantly (*p* < 0.05) higher in P4-FRD than in P4-ND and CT-FRD rats (Table 2). Interestingly, P4-primed rats already displayed higher (*p* < 0.05 *vs.* CT-ND values) plasma triacylglycerol levels when fed a ND (Table 2) and circulating levels of TG significantly (*p* < 0.05 *vs*. their respective ND group-values) increased after fructose administration in both groups (CT-FRD and P4-FRD) (Table 2), a parameter aggravated by P4 in FRD fed rats (*p* < 0.05 for P4-FRD *vs*. CT-FRD values, see Table 2). Similarly, priming rats with P4 (P4-ND rats) induced a significant (*p* < 0.05 *vs.* CT-ND values) rise in peripheral NEFA concentrations when rats were fed a ND, and intake of a FRD significantly (*p* < 0.05 *vs.* CT-ND values) increased peripheral NEFA concentrations in CT rats (Table 2). 

**Table 2:** Circulating levels of several metabolites in control (CT) and progesterone-primed (P4) rats examined 21 days after either ND or FRD intake. Values are means ± SEM, *n* = 6–7 rats per group.

	CT	P4
Glucose (mM)		
ND	6.33 ± 0.28	6.01 ± 0.33
FRD	6.72 ± 0.31	7.38 ± 0.36
Insulin (nM)		
ND	6.17 ± 1.53	6.94 ± 0.68
FRD	5.51 ± 0.61	5.39 ± 0.72
Total Cholesterol (mM)		
ND	1.64 ± 0.13	1.58 ± 0.08
FRD	1.34 ± 0.15	1.91 ± 0.12 ^b,c^
Triacylglycerol (mM)		
ND	0.99 ± 0.08	1.41 ± 0.09 ^a^
FRD	1.44 ± 0.22 ^a^	1.97 ± 0.18 ^c^
NEFA (mM)		
ND	0.35 ± 0.04	0.53 ± 0.06 ^a^
FRD	0.51 ± 0.05 ^a^	0.52 ± 0.07
Corticosterone (μM)		
ND	0.35 ± 0.06	0.45 ± 0.08
FRD	0.33 ± 0.09	0.43 ± 0.09
Progesterone (nM)		
ND	37.34 ± 8.14	48.31 ± 14.22
FRD	47.16 ± 9.51	31.36 ± 7.89
Estradiol (nM)		
ND	0.19 ± 0.03	0.20 ± 0.04
FRD	0.22 ± 0.02	0.26 ± 0.03
Testosterone (nM)		
ND	0.86 ± 0.06	0.98 ± 0.05
FRD	0.95 ± 0.08	0.91 ± 0.17

^a^ *p* < 0.05 *vs.* CT-ND values; ^b^*p* < 0.05 *vs.* P4-ND values; ^c^*p* < 0.05 *vs.* CT-FRD values.

**Figure 2:**
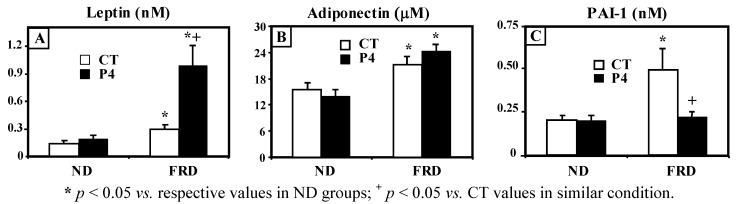
Leptin (**A**), adiponectin (**B**) and PAI-1 (**C**) plasma concentrations in CT and P4 rats fed a ND or FRD. Values are means ± SEM, *n* = 6–7 animals per group.

Regarding plasma adipokine levels, we found that leptin values were similar in CT-ND and P4-ND, and that they were significantly (*p* < 0.05) enhanced after FRD intake in both groups. However, the leptinemia attained by P4-FRD rats was even higher (*p* < 0.05) than that reached by CT-FRD rats (Figure 2A). While circulating adiponectin levels were similar in both ND groups, they were significantly (*p* < 0.05 *vs.* respective ND group-values) higher after FRD intake regardless of the group examined (Figure 2B). Conversely, although similar in both groups of rats fed a ND, plasma PAI-1 levels were significantly (*p* < 0.05 *vs.* CT-ND values) enhanced by FRD intake in CT but not in P4 rats (Figure 2C).

### 3.3. Changes in Plasma Glucose and Insulin Concentrations throughout the i.v.-GTT

**Figure 3:**
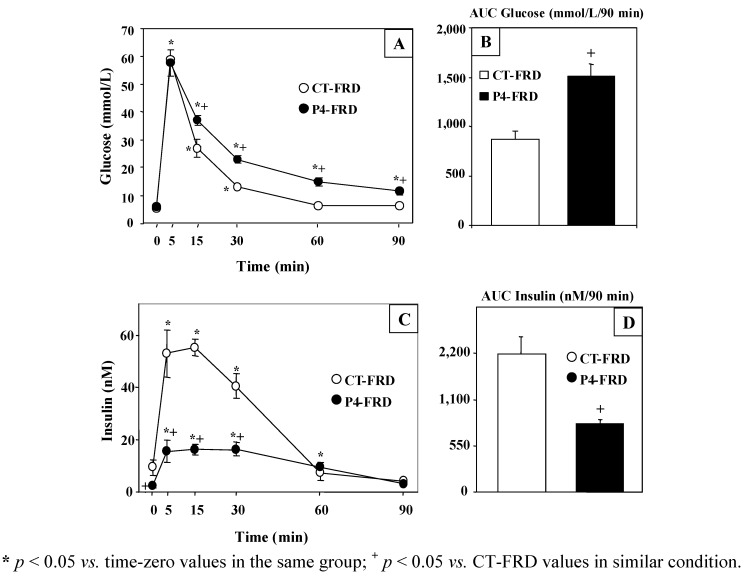
Circulating levels of glucose (**A**) and insulin (**C**) during the i.v.-GTT in CT and P4-treated rats fed a FRD. The area under the curve (AUC) throughout the test of circulating levels of glucose (**B**) and insulin (**D**) are also depicted. Values are means ± SEM, *n* = 6–7 animals per group.

Data from the i.v.-GTT indicate that although overnight fasting did not modify basal (time zero values) plasma glucose concentrations (Figure 3A), it significantly (*p* < 0.05 *vs.* CT-FRD values) reduced basal plasma insulin levels in P4-FRD rats (Figure 3C). After a high (2 g/kg BW) glucose load and despite similar plasma 5 min peak values of glucose in both groups examined, circulating glucose and insulin concentrations were significantly (*p* < 0.05) higher and lower in P4-FRD than in CT-FRD rats, respectively. Moreover, P4-FRD animals had not recovered basal plasma values of glucose by the end of the test (Figure 3A) and starting plasma insulin concentrations were reached 30 min later in P4-FRD than in CT-FRD rats (Figure 3C).

As expected, the areas under the curves (AUC) of plasma glucose (Figure 3B) and insulin (Figure 3D) concentrations throughout the test were significantly (*p* < 0.05) higher and lower in P4-FRD than in CT-FRD rats, respectively. 

In order to avoid any confusing effect of the earlier P4 treatment, additional studies were run on CT and P4 rats fed a ND (CT-ND and P4-ND rats). These data indicated that P4 treatment did not modify any parameter during the i.v.-GTT (*n* = 5–6 rats per group). In fact, overnight fasting-induced insulinemia was similar in both groups (2.39 ± 0.68 and 2.11 ± 0.59 nM; in CT-ND and P4 ND, respectively). Moreover, no group-differences were found in the AUC values of both glucose (973.03 ± 52.23 and 997.39 ± 98.57 mmol/L/90 min; in CT-ND and P4 ND, respectively) and insulin (2020.98 ± 406.61 and 1793.95 ± 411.24 nM/90 min; in CT-ND and P4-ND, respectively). 

### 3.4. Effects of P4 Treatment and FRD on *PMAT* Genes

**Figure 4:**
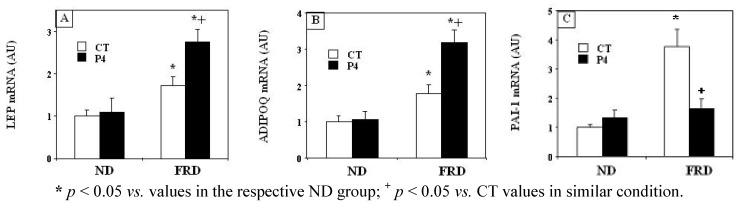
PMAT pad mRNA abundance of leptin (LEP; **A**), adiponectin (ADIPOQ; **B**) and PAI-1 (**C**) measured (by q-PCR) in different experimental groups. Values are expressed as the mean ± SEM (*n* = 4 pads per group).

PMAT leptin, adiponectin and PAI-1 mRNA expression levels (Figure 4A,B,C) were significantly (*p* < 0.05 *vs.* CT-ND group values) higher in CT-FRD rats. FRD administration in P4-treated rats also enhanced (*p* < 0.05 *vs.* P4-ND group-values) PMAT leptin and adiponectin mRNA abundance (Figure 4A,B). Moreover, these increases were even higher (*p* < 0.05) than the respective values found in CT-FRD group (Figure 4A,B). Conversely, administration of a FRD to P4-primed rats did not enhance PMAT PAI-1 mRNA expression, an effect fully independent of any previous change induced by P4 treatment (see CT-ND and P4-ND group-values in Figure 4C).

Finally, feeding rats a FRD did not induce any significant group-difference in the abundance of both GLUT-4 (in arbitrary units, AU; 1.09 ± 0.16 and 1.32 ± 0.32 in CT-FRD and P4-FRD groups respectively; *n* = 4 rats per group, *p* > 0.05) and IRS-1 (in AU; 1.07 ± 0.09 and 0.84 ± 0.13 in CT-FRD and P4-FRD groups respectively; *n* = 4 rats per group, *p* > 0.05) mRNA levels in PMAT pads. 

## 4. Discussion

In the present study we have shown for the first time that the metabolic, endocrine and adipose tissue dysfunction induced by short-term administration of a FRD to adult female rat can be prevented, at least partly, by previous P4 treatment. This P4 pretreatment unevenly affected the FRD-induced PMAT adipokine overproduction: whereas it enhanced leptin production and prevented PAI-1 production. We underscore that, unrelated to any intrinsic steroid effect, FRD rats treated with P4 had impaired glucose tolerance due to decreased high glucose-stimulated insulin secretion. Although the adipo-insular axis function in P4-FRD rats is well adapted in basal condition (*i.e*., normal plasma levels of glucose and insulin in non-fasting condition), with application of an allostatic load (e.g., high glucose load) the deleterious effect of P4 [15] on insulin release was observed.

Our current results support previously reported data related to metabolic, endocrine and AT dysfunction in normal adult female rats fed a FRD [5]. We now found that P4 administration to rats fed a standard diet (P4-ND) failed to modify plasma total cholesterol levels but enhanced those of TG and NEFA. Also, the FRD induced an increase in plasma cholesterol levels in P4-primed rats. These observations agree with previous data showing *in vivo* lipogenic activity of P4 in normal female rats [14], an effect ascribed to the ability of P4 to up-regulate fatty acid synthase activity [28]. Therefore, previous P4 administration could play a relevant role in the lipidic and endocrine dysfunctions induced by intake of a FRD. 

The changes recorded in PMAT endocrine function in rats fed a FRD strongly depend on the host to which this allostatic load was applied: whereas in normal (CT) rats the FRD-induced increase in leptin plasma levels was aggravated by previous P4 administration, increased circulating levels of PAI-1 was prevented by P4 treatment. Conversely, the FRD-elicited change in adiponectin production was similar in the two groups. 

It is accepted that the provision of an excess of a substrate (e.g., fructose) over a period of time increases NEFA production [29], AT OS [6,29] and several other AT dysfunctions [5,6,7]. Brownlee has postulated that substrate overload increases adipocyte-ROS mitochondrial production which results in multiple impairments, namely: (a) decreased antioxidant defense, (b) enhanced activity of protein kinase C isoform and PAI-1 production and (c) increased non-enzymatic glycosylation rate [30]. It has also been reported that the administration of a FRD induces OS that significantly affects AT [5,6,7] and other tissues functions [6,31]. Indeed, the FRD-elicited OS at the liver level triggers a local increase in glycogen storage, glucose-6-phosphate dehydrogenase activity, lactate production, glucokinase and glucose-6-phosphatase activities, thus leading to an increase in liver and plasma triacylglycerol levels [31]. All these changes might represent an adaptive defense mechanism against fructose overload in order to decrease substrate provision to the mitochondria, thus lowering ROS production. Also, the increased activity of the pentose pathway would provide the required NADPH for fatty acid synthesis, which then favors lipogenesis. Enhanced production of NADPH could also activate NADPH oxidase, thus establishing a vicious circle aggravating the overall OS induced by FRD intake. 

We previously reported that administration of a FRD to normal rats induced an increase in AT mass at the expense of the size of its adipocytes [5,7] associated with leptin overproduction [5]. The latter dysfunction was currently enhanced in animals pretreated with P4. Since high leptin levels impair insulin secretion and its activity [32], an overproduction of adiponectin [5,33] would be a crucial compensatory mechanism to overcome these alterations consecutive to the hyperleptinemia observed in P4-FRD rats. Although P4 produced in FRD rats all these detrimental effects on AT lipid metabolism and leptin production [34,35], it exerted a significant protective effect on the high PAI-1 production induced by this diet. In this regard, although little information exists, synthetic progestins have been shown to be effective in reducing PAI-1 production, both *in vitro* [36] and *in vivo* [37,38], thus reducing the high risk for developing cardiovascular disease in post-menopausal women. 

In summary, although the lipogenic effect of an excess of fructose intake throughout the diet remained/enhanced after previous P4 administration, our study supports that P4 effectively prevents the high PAI-1 production (plasma levels and AT mRNA concentration) induced by FRD administration to normal adult female rats. Our main finding is consistent with the previously described inverse relationship between endogenous PAI-1 production and peripheral progesterone concentration [39,40]. Because menopause heralds a drastic reduction in circulating levels of sex steroid hormones, this decline may contribute to increase cardiovascular risk by affecting adiposity, lipid metabolism and the prothrombotic state [41]. 

## 5. Conclusion

The present study supports the concept that P4 exerts a dual effect upon fat tissue metabolism: It enhances *in vivo* lipogenic activity, induced by administration of a FRD, and prevents the PAI-1 overproduction induced by this diet. However, we have yet to determine the precise mechanism whereby P4 exerts its modulatory effect upon nutrient-gene interaction and the potential partial adjustment of the nutrient-induced cell dys-metabolome [42].

## References

[1] Elliott S.S., Keim N.L., Stern J.S., Teff K., Havel P.J. (2002). Fructose, weight gain, and the insulin resistance syndrome. Am. J. Clin. Nutr..

[2] Bray G.A., Nielsen S.J., Popkin B.M. (2004). Consumption of high-fructose corn syrup in beverages may play a role in the epidemic of obesity. Am. J. Clin. Nutr..

[3] Kohen-Avramoglu R., Theriault A., Adeli K. (2003). Emergence of the metabolic syndrome in childhood: An epidemiological overview and mechanistic link to dyslipidemia. Clin. Biochem..

[4] Verma S., Bhanot S., Yao L., McNeill J.H. (1997). Vascular insulin resistance in fructose-hypertensive rats. Eur. J. Pharmacol..

[5] Alzamendi A., Castrogiovanni D., Ortega H.H., Gaillard R.C., Giovambattista A., Spinedi E. (2010). Parametrial adipose tissue and metabolic dysfunctions induced by fructose-rich diet in normal and neonatal-androgenized adult female rats. Obesity.

[6] Rebolledo O.R., Marra C.A., Raschia A., Rodríguez S., Gagliardino J.J. (2008). Abdominal adipose tissue: Early metabolic dysfunction associated to insulin resistance and oxidative stress induced by an unbalanced diet. Horm. Metab. Res..

[7] Alzamendi A., Giovambattista A., Raschia A., Madrid V., Gaillard R.C., Rebolledo O., Gagliardino J.J., Spinedi E. (2009). Fructose-rich diet-induced abdominal adipose tissue endocrine dysfunction in normal male rats. Endocrine.

[8] Delbosc S., Paizanis E., Magous R., Araiz C., Dimo T., Cristol J.P., Cros G., Azay J. (2005). Involvement of oxidative stress and NADPH oxidase activation in the development of cardiovascular complications in a model of insulin resistance, the fructose-fed rat. Atherosclerosis.

[9] Kumtepe Y., Borekci B., Karaca M., Salman S., Alp H.H., Suleyman H. (2009). Effect of acute and chronic administration of progesterone, estrogen, FSH and LH on oxidant and antioxidant parameters in rat gastric tissue. Chem. Biol. Interact..

[10] Behl C., Trapp T., Skutella T., Holsboer F. (1997). Protection against oxidative stress-induced neuronal cell death—A novel role for RU486. Eur. J. Neurosci..

[11] Perelló M., Castrogiovanni D., Giovambattista A., Gaillard R.C., Spinedi E. (2007). Impairment in insulin sensitivity after early androgenization in the post-pubertal female rat. Life Sci..

[12] Lorenzo M., Roncero C., Fabregat I., Benito M. (1988). Hormonal regulation of rat foetal lipogenesis in brown-adipocyte primary cultures. Biochem. J..

[13] Roncero C., Lorenzo M., Benito M. (1987). Regulation of rat foetal lipogenesis in brown adipose tissue *in vivo* and in isolated brown adipocytes during the last day of, and after prolonged, gestation. Biochem. J..

[14] Shirling D., Ashby J.P., Baird J.D. (1981). Effect of progesterone on lipid metabolism in the intact rat. J. Endocrinol..

[15] Sandberg M., Borg L.A. (2007). Steroid effects on intracellular degradation of insulin and crinophagy in isolated pancreatic islets. Mol. Cell. Endocrinol..

[16] Palomar-Morales M., Morimoto S., Mendoza-Rodríguez C.A., Cerbón M.A. (2010). The protective effect of testosterone on streptozotocin-induced apoptosis in beta cells is sex specific. Pancreas.

[17] Straub S.G., Sharp G.W., Meglasson M.D., de Souza C.-J. (2001). Progesterone inhibits insulin secretion by a membrane delimited, non-genomic action. Biosci. Rep..

[18] Ordóñez P., Moreno M., Alonso A., Fernández R., Díaz F., González C. (2007). Insulin sensitivity in streptozotocin-induced diabetic rats treated with different doses of 17beta-oestradiol or progesterone. Exp. Physiol..

[19] González C., Alonso A., Alvarez N., Díaz F., Martínez M., Fernández S., Patterson A.M. (2000). Role of 17beta-estradiol and/or progesterone on insulin sensitivity in the rat: Implications during pregnancy. J. Endocrinol..

[20] Sutter-Dub M.T., Dazey B., Hamdan E., Vergnaud M.T. (1981). Progesterone and insulin-resistance: Studies of progesterone action on glucose transport, lipogenesis and lipolysis in isolated fat cells of the female rat. J. Endocrinol..

[21] Ashby J.P., Shirling D., Baird J.D. (1981). Effect of progesterone on the secretion and peripheral action of insulin and glucagon in the intact rat. J. Endocrinol..

[22] Castrogiovanni D., Perelló M., Gaillard R.C., Spinedi E. (2003). Modulatory role of testosterone in plasma leptin turnover in rats. Endocrine.

[23] Perelló M., Castrogiovanni D., Moreno G., Gaillard R.C., Spinedi E. (2003). Neonatal hypothalamic androgenization in the female rat induces changes in peripheral insulin sensitivity and adiposity function at adulthood. Neur. Endocrinol. Lett..

[24] Spinedi E., Suescun M.O., Hadid R., Daneva T., Gaillard R.C. (1992). Effects of gonadectomy and sex hormone therapy on the endotoxin-stimulated hypothalamo-pituitary-adrenal axis: Evidence for a neuroendocrine-immunological sexual dimorphism. Endocrinology.

[25] Alzamendi A., Castrogiovanni D., Gaillard R.C., Spinedi E., Giovambattista A. (2010). Increased male offspring’s risk of metabolic-neuroendocrine dysfunction and overweight after fructose-rich diet intake by the lactating mother. Endocrinology.

[26] Chomczynski P., Sacchi N. (1987). Single-step method of RNA isolation by acid guanidinium thiocyanate-phenol-chloroform extraction. Anal. Biochem..

[27] Zar J.H. (1974). Biostatistical Analysis.

[28] Lacasa D., Le Liepvre X., Ferre P., Dugail I. (2001). Progesterone stimulates adipocyte determination and differentiation 1/sterol regulatory element-binding protein 1c gene expression. Potential mechanism for the lipogenic effect of progesterone in adipose tissue. J. Biol. Chem..

[29] Furukawa S., Fujita T., Shimabukuro M., Iwaki M., Yamada Y., Nakajima Y., Nakayama O., Makishima M., Matsuda M., Shimomura I. (2004). Increased oxidative stress in obesity and its impact on metabolic syndrome. J. Clin. Invest..

[30] Brownlee M. (2005). The pathobiology of diabetic complications: A unifying mechanism. Diabetes.

[31] Francini F., Castro M.C., Schinella G., García M.E., Maiztegui B., Raschia M.A., Gagliardino J.J., Massa M.L. (2010). Changes induced by a fructose-rich diet on hepatic metabolism and the antioxidant system. Life Sci..

[32] Walder K., Filippis A., Clark S., Zimmet P., Collier G.R. (1997). Leptin inhibits insulin binding in isolated rat adipocytes. J. Endocrinol..

[33] Berg A.H., Combs T.P., Du X., Brownlee M., Scherer P.E. (2001). The adipocyte-secreted protein Acrp30 enhances hepatic insulin action. Nat. Med..

[34] Withers D.J., White M. (2000). Perspective: The insulin signaling system—A common link in the pathogenesis of type 2 diabetes. Endocrinology.

[35] Krotkiewski M., Björntorp P. (1976). The effect of progesterone and of insulin administration on regional adipose tissue cellularity in the rat. Acta Physiol. Scand..

[36] Mueck A.O., Lippert C., Seeger H., Wallwiener D. (2003). Effects of tibolone on human breast cancer cells and human vascular coronary cells. Arch. Gynecol. Obstet..

[37] Winkler U.H., Altkemper R., Kwee B., Helmond F.A., Coelingh Bennink H.J. (2000). Effects of tibolone and continuous combined hormone replacement therapy on parameters in the clotting cascade: A multicenter, double-blind, randomized study. Fertil. Steril..

[38] Koh K.K., Han S.H., Shin M.S., Ahn J.Y., Lee Y., Shin E.K. (2005). Significant differential effects of lower doses of hormone therapy or tibolone on markers of cardiovascular disease in post menopausal women: A randomized, double-blind, crossover study. Eur. Heart J..

[39] Willeit J., Kiechl S. (2000). Biology of arterial atheroma. Cerebrovasc. Dis..

[40] Liu Y.X. (2004). Plasminogen activator/plasminogen activator inhibitors in ovarian physiology. Front. Biosci..

[41] Schneider J.G., Tompkins C., Blumenthal R.S., Mora S. (2006). The metabolic syndrome in women. Cardiol. Rev..

[42] Go V.L., Nguyen C.T., Harris D.M., Lee W.N. (2005). Nutrient-gene interaction: Metabolic genotype-phenotype relationship. J. Nutr..

